# Cox’s Companion to the Sea Medicine Chest, and Compendium of Domestic Medicine; Particularly Adapted for Captains of Merchant Vessels, &c., with Plain Rules for Taking the Medicines; Directions for Restoring Suspended Animation, the Method of Obviating the Effect of Poisons, &c., &c.

**Published:** 1852-01

**Authors:** 


ART. IX.—*Cox's Companion to the Sea Medicine Chest*, and Compendium of Domestic Medicine; particularly adapted for Captains of Merchant Vessels, &c., with plain rules for taking the medicines; directions for restoring suspended animation, the method of obviating the effect of poisons, &c., &c. Revised and considerably enlarged by R. DAVIS, Member of the Royal College of Surgeons. First American from the thirty-third London edition. New York. Samuel S. & William Wood. 1851. 12mo. pp. 276.

An American edition of Cox's Companion is an acceptable addition to the literature and resources of popular medicine. This edition has also useful additions of many new articles, and the vulgar names of medicines before given only with their technical designation.

"The circumstances of this little work having passed through thirty-two editions is a sufficient guarantee, if any were wanting, of its value, and of the estimation in which it has been held by the public far so many years."

"The book, as it now stands, is divided into three departments or divisions:—First, a PHARMACEUTICAL or DRUG DEPARTMENT, in which is contained a full account of all the principal medicines in use, their properties, qualities, and doses, as administered in the cure of disease. Secondly, a SURGICAL DEPARTMENT, in which is given a concise description of all the more common dislocations and fractures incidental to persons employed in laborious occupations, together with their symptoms and treatment. Thirdly, a MEDICAL DEPARTMENT, which points out the best mode of treating successfully those bodily complaints to which children and grown persons are subject. The whole is written in an easy style, and free from all technicalities, so that he that runs may not only read but understand.

In the commencement of the work will be found a SCALE OF DOSES, by which means the proper dose of any medicine suited to different ages will be seen at a glance, and an Alphabetical List of the drugs spoken of; and, at the end, a List of the Medical Terms used in the work, with their explanation in English, as well as a copious Index, by which any given subject may be immediately found without delay."—*Preface.*

